# The zebrafish Kupffer's vesicle as a model system for the molecular mechanisms by which the lack of Polycystin-2 leads to stimulation of CFTR

**DOI:** 10.1242/bio.014076

**Published:** 2015-10-02

**Authors:** Mónica Roxo-Rosa, Raquel Jacinto, Pedro Sampaio, Susana Santos Lopes

**Affiliations:** CEDOC, Chronic Diseases Research Center, NOVA Medical School/Faculdade de Ciências Médicas, Universidade Nova de Lisboa, Campo dos Mártires da Pátria, 130, Lisboa 1169-056, Portugal

**Keywords:** Autosomal dominant polycystic kidney disease, (ADPKD), Cystic fibrosis transmembrane conductance regulator, (CFTR), Kupffer's vesicle, (KV), Polycystin-2, Zebrafish

## Abstract

In autosomal dominant polycystic kidney disease (ADPKD), cyst inflation and continuous enlargement are associated with marked transepithelial ion and fluid secretion into the cyst lumen via cystic fibrosis transmembrane conductance regulator (CFTR). Indeed, the inhibition or degradation of CFTR prevents the fluid accumulation within cysts. The *in vivo* mechanisms by which the lack of Polycystin-2 leads to CFTR stimulation are an outstanding challenge in ADPKD research and may bring important biomarkers for the disease. However, hampering their study, the available ADPKD *in vitro* cellular models lack the three-dimensional architecture of renal cysts and the ADPKD mouse models offer limited access for live-imaging experiments in embryonic kidneys. Here, we tested the zebrafish Kupffer's vesicle (KV) as an alternative model-organ. KV is a fluid-filled vesicular organ, lined by epithelial cells that express both CFTR and Polycystin-2 endogenously, being each of them easily knocked-down. Our data on the intracellular distribution of Polycystin-2 support its involvement in the KV fluid-flow induced Ca^2+^-signalling. Mirroring kidney cysts, the KV lumen inflation is dependent on CFTR activity and, as we clearly show, the knockdown of Polycystin-2 results in larger KV lumens through overstimulation of CFTR. In conclusion, we propose the zebrafish KV as a model organ to study the renal cyst inflation. Favouring its use, KV volume can be easily determined by *in vivo* imaging offering a live readout for screening compounds and genes that may prevent cyst enlargement through CFTR inhibition.

## Abstract (precis)

**Summary:** Here, we tested the zebrafish Kupffer's vesicle (KV) as a model organ to study, through *in vivo* imaging of KV volume, the stimulation of cystic fibrosis transmembrane conductance regulator (CFTR) in autosomal dominant polycystic kidney disease ADPKD.

## INTRODUCTION

The major clinical manifestation of autosomal dominant polycystic kidney disease (ADPKD) is the development of massive fluid-filled kidney cysts that destroy the renal parenchyma. ADPKD has no treatment and no biomarkers to predict renal function decline. Nowadays, patients are still managed with supportive measures only, namely analgesics, antibiotics for cyst infection, blood pressure control and avoidance of caffeine and oestrogens. About 50% of ADPKD patients reach end-stage renal disease by age 60, requiring dialysis and renal replacement therapy (Patel et al., 2009; Wallace, 2011).

ADPKD is the most common genetic cause and the fourth leading cause of kidney failure, affecting 1 in 400–1000 newborns. Mutations in the genes encoding Polycystin-1 (*pkd1*, OMIM-601313) and Polycystin-2 (*pkd2*, OMIM-613095) in Online Mendelian Inheritance of Man (http://www.ncbi.nlm.nih.gov/omim), account for all known forms of the disease (Cornec-Le Gall et al., 2013). Both genes present high level of allelic heterogeneity, with hundreds of reported mutations expected to range from loss of function to hypomorphic variants (http://pkdb.mayo.edu/). Polycystin-1 and 2 are transmembrane glycoproteins of 4302 and 968 amino acids, respectively. While Polycystin-1 has structural features of a mechanosensor (Hughes et al., 1995), its binding partner Polycystin-2 is a non-selective Ca^2+^-conducting channel (González-Perrett et al., 2001). These are expected to assemble together into a mechanosensor-channel complex in the membrane of primary cilia of kidney epithelial cells (Nauli et al., 2003). Although the precise function of this complex is still unresolved, it has been suggested that the urine-flow is sensed by Polycystin-1 and transduced into a ciliary Ca^2+^-signal by Polycystin-2 (Nauli et al., 2003; Praetorius and Spring, 2001). As shown by *in vitro* experiments, this ciliary Ca^2+^ wave is propagated and amplified in the cytoplasm, a process that depends on Polycystin-2 expressed at the endoplasmic reticulum (ER) (Jin et al., 2014; Nauli et al., 2003; Praetorius and Spring, 2001; Xu et al., 2007). The role of Polycystin-2 in Ca^2+^ homeostasis is essential for the differentiation and maintenance of the kidney tubular epithelium. Once disrupted, as in ADPKD cells, a reduction in basal intracellular Ca^2+^ levels occurs, which is thought to trigger cystogenesis (Spirli et al., 2012; Yamaguchi et al., 2006).

ADPKD cysts are anatomically separated from the tubule from which they derive (Grantham et al., 1987). While the normal renal tubule epithelium has mainly absorptive properties (for glomerular-filtrated reabsorption), cyst-lining cells have abnormally high capacity of ion and fluid secretion into the cyst lumen. This entails marked modifications in the activity of ion-channels. Activation of cystic fibrosis transmembrane conductance regulator (CFTR) plays a central role in this process. Triggering Cl^−^ and water secretion towards the cyst lumen, CFTR promotes cyst inflation (Torres and Harris, 2014). As it is a key Cl^−^ channel for regulating epithelial ion and fluid transport, the loss of CFTR (OMIM-602421) underlies cystic fibrosis (CF), a recessive disease characterized by mucus build-up in several organs (Amaral and Farinha, 2013). In contrast to ADPKD, CF patients do not have major renal problems. Perhaps because kidney tissue from healthy individuals have low levels of CFTR (Hanaoka et al., 1996), also supported by the evidence that ectopic expression of Polycystin-1 reduces its apical expression in mammalian kidney cells (Ikeda et al., 2006). Highlighting its involvement in ADPKD, Hanaoka et al*.* reported a strong expression of CFTR in cyst-lining cells from ADPKD patients and shown that fluid accumulation within cysts involves CFTR-like Cl^−^ currents (Hanaoka et al., 1996). Further evidence comes from the cyst inflation being slowed down either through pharmacological inhibition of CFTR or by reducing its apical expression in cyst-lining cells (Blazer-Yost et al., 2010; Li et al., 2004, 2012; Yang et al., 2008; Yuajit et al., 2013, 2014). Also, a milder renal disease was observed in few patients affected simultaneously by ADPKD and CF (Xu et al., 2006).

Not much is known about the molecular mechanisms behind the activation of CFTR in cyst epithelial cells. Converging data support the hypothesis that it may result from the impairment of the 3′,5′-cyclic adenosine monophosphate (cAMP) homeostasis. Increased intracellular cAMP levels are among the most consistently described changes associated with ADPKD. Indeed, vasopressin and forskolin exacerbate renal cyst growth via increasing intracellular cAMP levels (Wallace, 2011). In contrast, Tolvaptan, a vasopressin V2 receptor antagonist, slows down cystogenesis by lowering the levels of this second messenger (Reif et al., 2011; Torres et al., 2012). It is thought that the combination of increased production and decreased degradation of cAMP raises its basal concentration to levels closer to the threshold for Protein kinase A (PKA) activation, leading to CFTR stimulation. Indeed, CFTR stimulation requires its prior cAMP-dependent phosphorylation by PKA (Amaral and Farinha, 2013).

The role of CFTR in ADPKD has been approached using *in vitro* cellular models such as Madin–Darby canine kidney (MDCK) cells (Li et al., 2004, 2012; Yuajit et al., 2013) and ADPKD cyst-derived cell lines (Reif et al., 2011). These cellular models form cyst-like structures when grown in a collagen matrix, lacking, however, the three-dimensional architecture of renal cysts. The effectiveness of CFTR-interfering drugs in preventing cystogenesis has also been demonstrated using ADPKD mouse models (Blazer-Yost et al., 2010; Yang et al., 2008; Yuajit et al., 2014). However, the limited access to the kidneys of mouse embryos at early developmental stages, namely for live-imaging experiments, hinders their widespread use to study the precise mechanisms underlying the CFTR stimulation.

The zebrafish mutant for the orthologous gene of human *pkd2*, the *curly-up* (*cup^−/−^*) mutant has emerged as a model-organism to study cardiovascular problems (Paavola et al., 2013), organ laterality (Schottenfeld et al., 2007) and midline axis defects (Le Corre et al., 2014). However, limiting its usage to study the ADPKD cystogenesis, *cup^−/−^* mutants do not develop kidney cysts, probably because of the maternal contribution of *pkd2* mRNA present during early embryonic stages (Schottenfeld et al., 2007; Sun et al., 2004). Alternatively, the injection of morpholinos against *pkd2* mRNA to specifically knockdown its translation from one-cell stage induces pronephric cysts (Obara et al., 2006; Schottenfeld et al., 2007; Sun et al., 2004). However, while impairing the fluid homeostasis of the animal, these cysts were mainly pronephric dilations that never get to bud off from the tubules, thus, not recapitulating the vesicular architecture of the ADPKD patients' cysts.

**Fig. 1.**
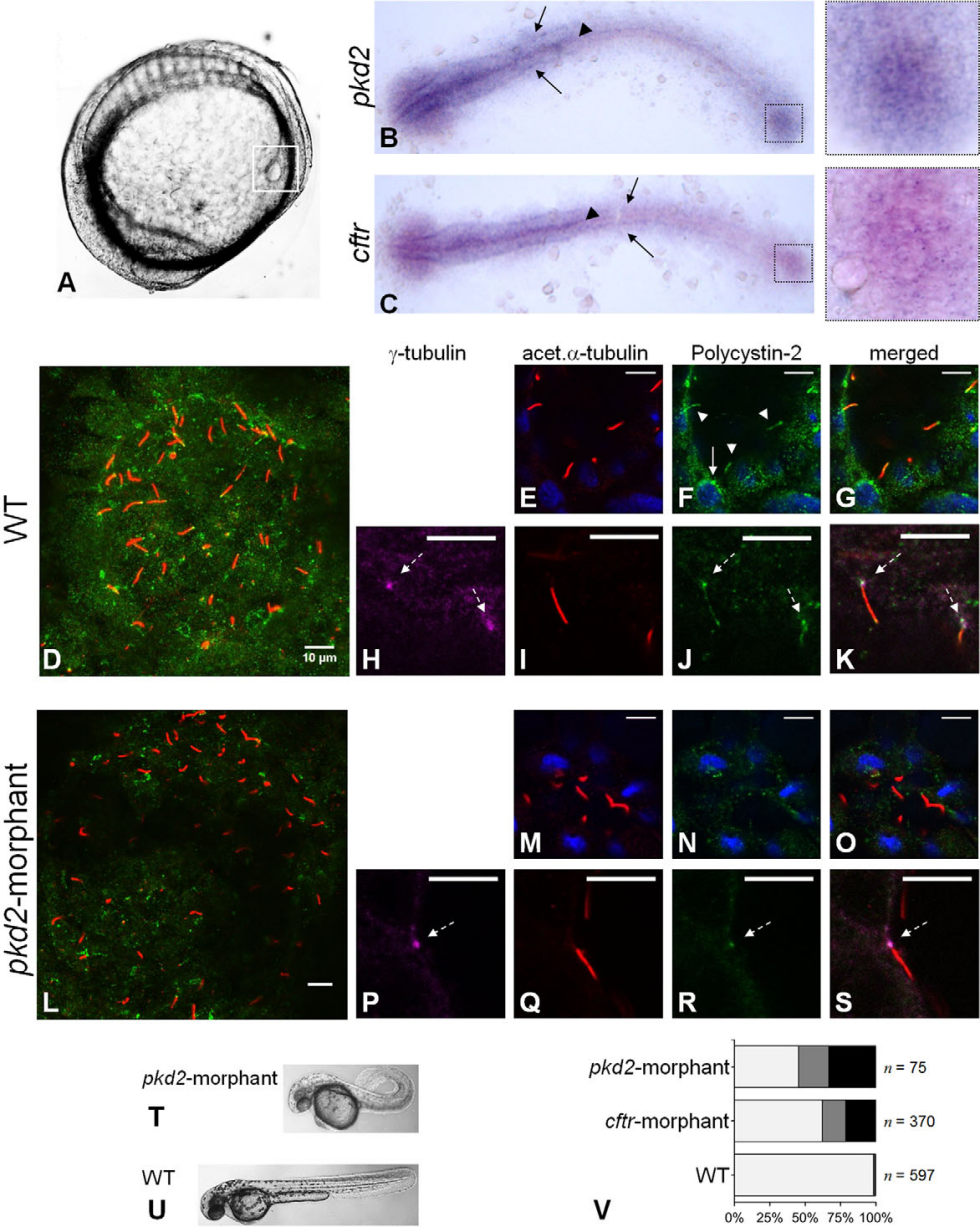
**Polycystin-2 and CFTR expression.** (A) Localization of KV (squared region) in the body of a 10–11 s.s. zebrafish embryo. (B,C) RNA *in situ* hybridizations for *pkd2* (B) and *cftr* (C) in 10–11 s.s. WT embryos. Both *pkd2* and *cftr* transcripts are detected in the KV region (right squares), neural floorplate (arrow heads), brain and pronephric ducts primordia (arrows). (D-S) Confocal images for the immunolocalization of Polycystin-2 in KV cells at the 10–11 s.s. In WT embryos (D-K), Polycystin-2 is detected clustered near the nuclei (white arrow in F), along cilia (white arrow heads in F) and at the basal body (dashed arrows in H,J and K). In *pkd2*-morphants (L-S), the Polycystin-2 signal is markedly reduced and, although still detected at the basal body (dashed arrow in P,R and S), it is no longer detected along cilia. (D,L) maximal intensity *z*-stack projection; (E-K;M-S) *z*-section. Polycystin-2 (green), acetylated α-tubulin (red), γ-tubulin, (purple), nuclei (blue). Scale bars: 10 µm. (T,U) Lateral view of *pkd2*-morphant (T) and WT (U) larvae at 72 hpf. (V) Heart position defects: *pkd2-*morphants – 33% right-sided, 21% central; *cftr-*morphants – 21% right-sided, 17% central; and WT siblings – 0.7% right-sided, 1.0% central. Left-sided (light grey), central (dark grey) and right-sided hearts (black). *n*, number of scored embryos.

Aiming to overcome the imperfect fish model, we investigated the usefulness of zebrafish Kupffer's vesicle (KV) as a model organ for kidney cyst inflation. The KV is an organ transiently present in the normal early embryonic life of the fish to establish internal body laterality (Essner et al., 2005; Sampaio et al., 2014; Smith et al., 2014). It derives from the dorsal forerunner cells (DFCs) that cluster at the tailbud of the embryo (Essner et al., 2005; Sampaio et al., 2014; Smith et al., 2014). Although not being a renal-related organ, the KV is a fluid-filled vesicle (Fig. 1A) which inflation depends on CFTR (Navis et al., 2013). It is lined by epithelial cells that express both CFTR (Navis et al., 2013) and, as we show here, Polycystin-2. While not affecting cell proliferation which is a marked difference to the renal cyst formation, we demonstrate that *pkd2*-knockdown causes a significant increase in the CFTR-mediated fluid-secretion into the KV lumen mirroring the cyst inflation process. We, thus, propose the zebrafish KV as a model organ to study the stimulation of CFTR in ADPKD.

## RESULTS

### *pkd2* and *cftr* are expressed in KV-lining cells

Previous studies have reported the presence of *pkd2* and *cftr* transcripts in the early stages of the zebrafish embryonic development (Bisgrove et al., 2005; Navis et al., 2013; Obara et al., 2006; Schottenfeld et al., 2007). Our *in situ* hybridization experiments, using different mRNA probes from those used in the mentioned studies, showed that at the 10–11somite stages (s.s.) *pkd2* and *cftr* are expressed in the same tissues of the embryo. Corroborating those studies, we detected both transcripts in the KV region (Fig. 1B,C) (Bisgrove et al., 2005; Navis et al., 2013; Schottenfeld et al., 2007). We also detected *pkd2* expression in the brain and in the neural floorplate as before (Bisgrove et al., 2005; Schottenfeld et al., 2007) and, less intensely, in the primordia of the pronephric ducts (Fig. 1B). Interestingly, we show here an identical pattern of expression for *cftr* transcripts (Fig. 1C).

To characterize the expression of Polycystin-2 in zebrafish KV, we performed whole-mount immunostaining in 10–11 s.s. wild-type (WT) embryos. Polycystin-2 was detected through the KV-lining cells, with a punctate pattern of expression (Fig. 1D), many times clustered near the nuclei (Fig. 1F). Given the similarity to the pattern described for Polycystin-2 in zebrafish pronephros (Fu et al., 2008; Obara et al., 2006) and in mouse (Gainullin et al., 2015; Pazour et al., 2002) and human kidney cells (Cai et al., 1999; Gainullin et al., 2015; Hofherr et al., 2014), we speculate that these clusters may correspond to the Polycystin-2 expressed at the ER membrane. We also found Polycystin-2 along the zebrafish KV cilia, co-localizing with acetylated α-tubulin (Fig. 1E-G), in complete agreement to the ciliary expression described for medaka KV cells (Kamura et al., 2011) and for zebrafish, mouse and human renal cells (Gainullin et al., 2015; Obara et al., 2006; Pazour et al., 2002). Obara et al*.* have also suggested the presence of Polycystin-2 at the base of zebrafish pronepheric cilia (Obara et al., 2006). Here, we clearly show that, indeed, Polycystin-2 is expressed at the basal body of the KV cilia, co-localizing with γ-tubulin (Fig. 1H-K).

Supporting the efficient knockdown of Polycystin-2, we were able to reduce its immunodetection by injecting one-cell stage embryos with 1.8 ng of *pkd2*-augMO (Fig. 1L). Indeed, the Polycystin-2 signal along the cilia was totally abolished (Fig. 1M-O) and, although we still detected it at the base of the cilia (Fig. 1P-S), its cytoplasmic signal was considerably lowered (Fig. 1L-O). This reduction was sufficient to induce a curly-up tail phenotype (Fig. 1T,U) and heart position defects (54% of right-sided or central hearts) (Fig. 1V), comparable to those obtained by others (Schottenfeld et al., 2007).

Regarding CFTR, Navis et al*.* have recently described a strong expression of this protein at the apical membrane of the cells facing the lumen of the zebrafish KV (Navis et al., 2013).

### *pkd2-*knockdown impacts on the KV volume

**Fig. 2.**
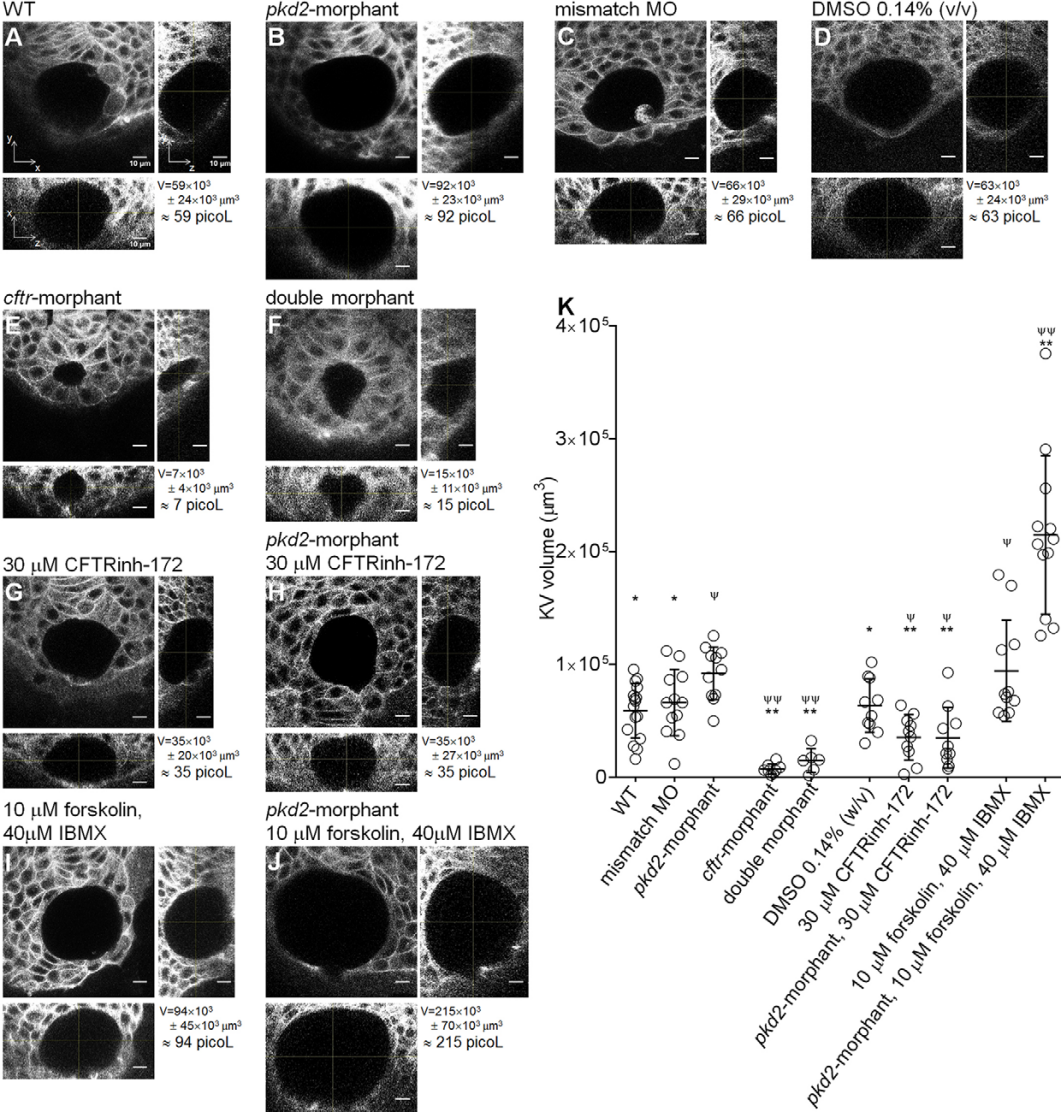
**KV volume.** (A-J) Confocal live-microscopy scans of the whole KV of 10–11 s.s. *ras*:GFP transgenic embryos. The middle focal plane along the *xy* axis and the respective orthogonal views (along *xz* and *yz* axes) are shown for the most representative WT (A), *pkd2*-morphant (B), *pkd2*-mismatch MO (C), 0.14% (v/v) DMSO-treated WT (D), *cftr*-morphant (E), double-morphant (F), 30 µM CFTRinh-172-treated WT (G) and *pkd2*-morphant (H), and 10 µM forskolin+40 µM IBMX-treated WT (I) and *pkd2*-morphant (J) embryos. C is a control for B. D is a control for G,H,I and J. KV^volume^ is indicated in µm^3^ and in picol. Scale bars: 10 µm. (K) Estimated KV volumes (µm^3^) for WT (*n=*16), *pkd2*-mismatch MO (*n=*12), *pkd2*-morphant (*n=*11), *cftr*-morphant (*n=*8), double-morphant (*n=*6), 0.14% (v/v) DMSO-treated WT (*n=*10), 30 µM CFTRinh-172-treated WT (*n=*10) and *pkd2*-morphant (*n=*10), 10 µM forskolin+40 µM IBMX-treated WT (*n*=11) and *pkd2*-morphant (*n=*12) embryos. Mean±s.d.; ^ψ^*P*≤0.05 and ^ψψ^*P*<0.0001, significantly different from WT; *P<0.05 and **P<0.0001, significantly different from *pkd2*-morphants.

In order to determine whether Polycystin-2 and CFTR have a similar relationship in the KV-lining cells to that observed in ADPKD cysts, we determined the impact of the knockdown of *pkd2* on the KV volume. For that, we scanned the whole KV by confocal live-microscopy of *ras*:GFP transgenic embryos at the 10–11 s.s. As demonstrated by the middle focal plan and respective orthogonal views, *pkd2*-morphants presented KVs with significant larger dimensions than their WT siblings or *pkd2*-mismatch MO injected embryos (Fig. 2A,B,C). On average *pkd2*-morphants presented KVs with 1.6 times the volume of WT siblings and 1.4 times the volume of the *pkd2*-mismatch MO injected controls (*pkd2*-morphant KV^volume^=92×10^3^±23×10^3^ µm^3^ vs WT KV^volume^=59×10^3^±24×10^3^ µm^3^ with *P*=0.0017 or vs *pkd2*-mismatch MO KV^volume^=66×10^3^±29×10^3^ µm^3^ with *P*=0.0289) (Fig. 2K). WT and *pkd2*-mismatch MO KVs were equivalent (Fig. 2A,C,K). Similar results were obtained in embryos at the 8–9 s.s. (data not shown).

**Fig. 3.**
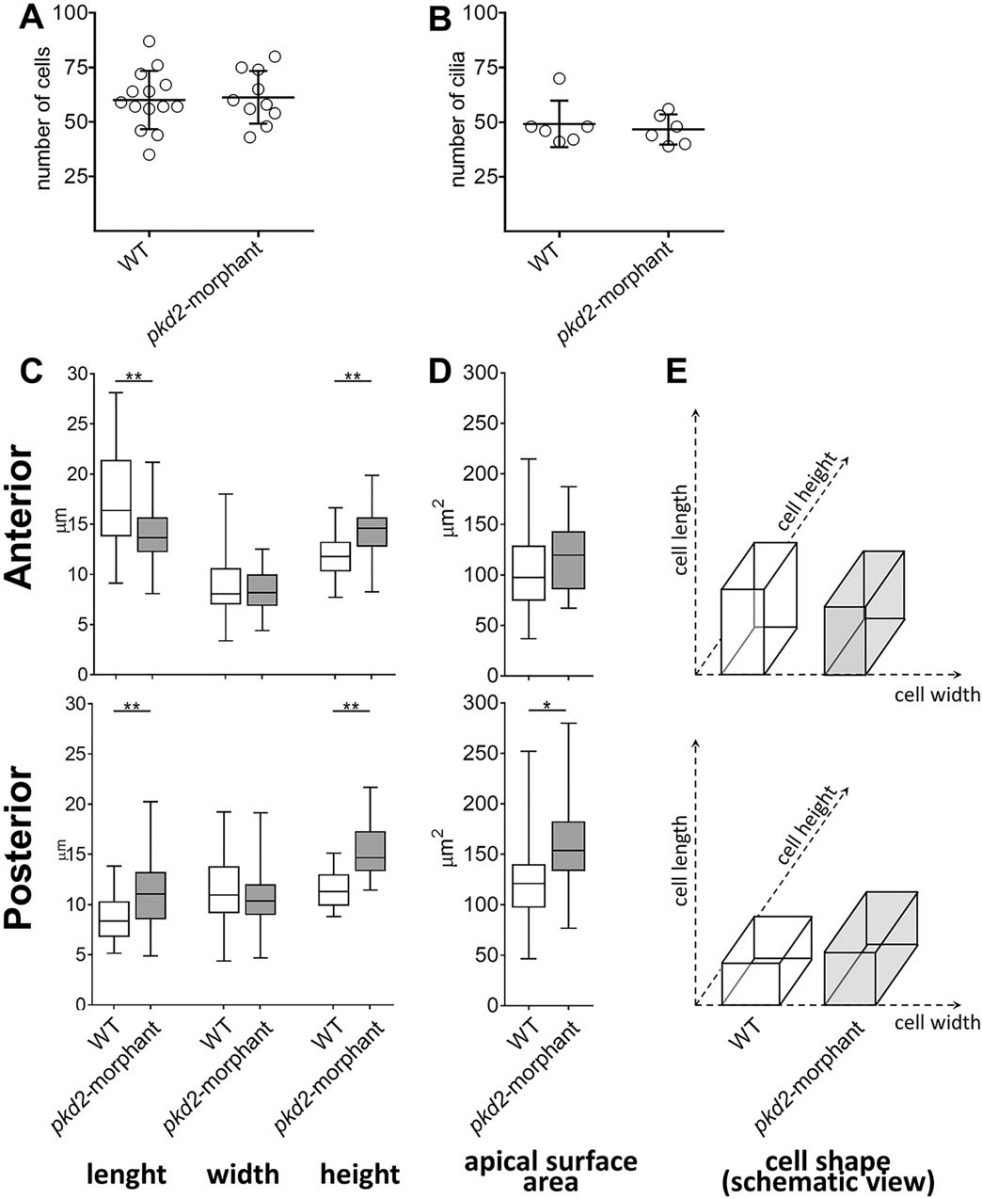
**KV-lining cells.** (A) Number of cells counted in the whole KV live-microscopy scans of 10–11 s.s. WT (*n=*14) and *pkd2*-morphant (*n=*10) embryos. (B) Number of cilia counted in WT (*n=*6) and *pkd2*-morphant (*n=*6) embryos immunodetected for acetylated α-tubulin. (C) Cellular length, width and height of WT (*n=*6) and *pkd2*-morphant (*n=*11) embryos immunodetected for actin cytoskeleton. The box plot and the respective max and min values are indicated. *P<0.01 and **P<0.0001, significantly different from WT. (D) Estimated apical surface area of KV-lining cells. (E) Schematic representation of cell shape of WT and *pkd2*-morphant KVs.

To determine the cause behind this observation, we counted the number of cells and cilia lining the KV of *pkd2*-morphants. As both parameters were equivalent to those of their WT siblings (Fig. 3A,B), we demonstrated that the knockdown of *pkd2* was not affecting KV cell proliferation. Therefore, our data were suggesting that reduction of Polycystin-2 protein levels drives increased fluid secretion into the KV lumen. In that case, changes in the shape of the cells facing the KV lumen would be expected. Indeed, the growth of the KV lumen through enhanced fluid secretion increases the KV intraluminal pressure and drives regional cell shape modifications (Compagnon et al., 2014). To clarify this, we assessed the shape of the cells from the anterior and posterior regions of *pkd2-*morphant and WT KVs (Fig. 3C-E). While not affecting the cells' width, the knockdown of *pkd2* significantly increased the cells' height both anteriorly and posteriorly (Fig. 3C). Moreover, it also affected significantly the cells' length. Thus, cells became shorter at the KV anterior part and longer at the posterior region (Fig. 3C,E). Supporting our hypothesis, those differences are translated in the increase of the apical surface of the cells facing the KV lumen posteriorly (*P*<0.01) (Fig. 3D).

We also assessed the KV volume of the progeny of the *cup^+/−^*;*foxj1a*:GFP parents. At the 10–11 s.s., the *foxj1a*:GFP transgene allows the detection of KV-lining cells (Fig. S1). The *cup^tc321^* is likely to be a null mutation and, when in homozygosity, it causes severe curly-up tail phenotypes and organ laterality defects, limiting the larvae survival (Schottenfeld et al., 2007). In our experiments, after having imaged the KVs from the progeny of the *cup^+/−^*;*foxj1a*:GFP parents, we allowed the embryos to grow until 72 hpf (hours post-fertilization) to distinguish the *cup^−/−^* mutants by their curly-up tails. On average the curly-up tail embryos presented KVs that were larger than their normal tail siblings (Fig. S1A,B). However, this difference was not statistically significant, nor as pronounced as that observed when comparing *pkd2*-morphant with WT siblings (Fig. S1C). This can be easily explained by the maternal contribution of *pkd2* mRNA previously detected in the early stages of the *cup* mutants' embryonic life (Schottenfeld et al., 2007). Corroborating this data, our whole-mount immunostaining experiments showed that, although in a less extent than in their normal tail siblings (Fig. S1D-G), at 36 hpf Polycystin-2 is still present near the basal body and along the pronephric cilia from curly up tail *cup*^−/−^ mutants (Fig. S1H-J). This effect must be undoubtedly more pronounced at earlier stages of development, during the KV life time. Also masking the differences in terms of KV volume is the fact that the group of normal tail embryos displays more variability because it gathers both *cup^+/−^* and *cup^+/+^* siblings. In conclusion, the *cup* mutant zebrafish line is not useful for the purpose of this work. A maternal zygotic may be considered as an alternative for future experiments.

### CFTR has a role in the enlargement of the KV after *pkd2*-knockdown

Navis et al. have described that CFTR is essential for the proper KV inflation (Navis et al., 2013). Efficiently phenocoping *cftr*-mutants, injection of 1.6 ng of *cftr*-augMO severely impaired the lumen expansion of the KV (*cftr*-morphants KV^volume^=7×10^3^±4×10^3^ µm^3^ vs WT KV^volume^=59×10^3^±24×10^3^ µm^3^; *P*<0.0001) (Fig. 2A,E,K). Thus, presenting KVs with 12% of the volume of their WT siblings (Fig. 2E), *cftr*-morphants have KV functional problems that justify the observed heart position defects (38% of right-sided or central hearts) (Fig. 1V).

In order to test whether the enlargement of the KV volume of the *pkd2*-morphants was reflecting an enhancement of the CFTR-dependent fluid secretion, we injected embryos at their one-cell stage with both morpholinos (*pkd2*-augMO and *cftr*-augMO) expecting to neutralize that effect. We tried to titrate the amount of *cftr*-augMO in order to bring the KV luminal volume of double-morphants to WT values. However, by injecting as low as 0.1 ng (14 times less than in *cftr*-morphants), we still observed an almost complete failure of KV lumen expansion (Fig. 2F,K). This suggests that the downregulation of *cftr* overrides the effect of downregulating *pkd2*.

This prompted us to address the role of CFTR in the KV inflation of the *pkd2*-morphants by pharmacological manipulation of the CFTR activity. Indeed, Navis et al*.* showed that the KV luminal area can be regulated by the pharmacological modulation of CFTR activity (Navis et al., 2013). We treated embryos with 5 µM ouabain solution from 6 to 10 s.s., a shorter time window than that used by those authors. WT embryos under this treatment presented a non-significant reduction in their KV volume (ouabain treated WT KV^volume^=44×10^3^±24×10^3^ µm^3^ vs WT KV^volume^=59×10^3^±24×10^3^ µm^3^; *P*>0.05) (Fig. S2). On the other hand, when we treated *pkd2*-morphant embryos using the same conditions, this was sufficient to reduce the volume of their KVs to WT values, i.e. significantly lower than those of non-treated *pkd2*-morphants (ouabain treated *pkd2*-morphants KV^volume^=56×10^3^±26×10^3^ µm^3^ vs *pkd2*-morphants KV^volume^=92×10^3^±23×10^3^ µm^3^; *P*=0.0013) (Fig. S2). However, a major drawback of ouabain is the fact that it is not specific for CFTR. Actually, this molecule exerts its action on CFTR by inhibiting Na^+^/K^+^-ATPase, a pump that supports the ion gradient between the intra- and the extracellular space, which is essential for CFTR activity (Lingrel, 2010). The inhibition of this central ATPase is, therefore, highly promiscuous, potentially affecting nearly all cellular transport. Therefore, we gave a step forward and tested the effect of a specific inhibitor of CFTR, thiazolidinone (CFTRinh-172), which was shown to slow down *in vitro* cyst enlargement (Li et al., 2004). By treating WT embryos with 30 µM CFTRinh-172 solution from 6 s.s. onwards, a significant reduction in their KV volume was observed (CFTRinh-172 treated WT KV^volume^=35×10^3^±20×10^3^ µm^3^ vs WT KV^volume^=59×10^3^±24×10^3^ µm^3^; *P*=0.0132) (Fig. 2A,G,K). Interestingly, *pkd2*-morphant embryos under the same conditions presented an even more pronounced reduction of their KV luminal volume. This was significantly lower than those of non-treated *pkd2*-morphants (CFTRinh-172 treated *pkd2*-morphants KV^volume^=35×10^3^±27×10^3^ µm^3^ vs *pkd2*-morphants KV^volume^=92×10^3^±23×10^3^ µm^3^; *P*<0.0001) and those of WT embryos (CFTRinh-172 treated *pkd2*-morphants vs WT KV^volume^=59×10^3^±24×10^3^ µm^3^; *P*=0.0034) (Fig. 2A,B,H,K). DMSO 0.14% (w/v) had no effect in KV volume (Fig. 2D,K).

To activate CFTR, we treated embryos with a cocktail of 10 µM forskolin+40 µM IBMX from 6 to 10 s.s., also a shorter time window than that used by Navis et al. (2013). The results showed that forskolin+IBMX treated WT embryos presented a significant increase in the KV volume, showing on average KVs with 1.6 times the volume of WT siblings (forskolin+IBMX treated WT embryos KV^volume^=94×10^3^±45×10^3^ µm^3^ vs WT KV^volume^=59×10^3^±24×10^3^ µm^3^; *P=*0.00321) (Fig. 2A,I,K). It is important to mention that Navis et al*.* showed that forskolin+IBMX failed to rescue the KV lumen expansion of zebrafish embryos homozygous for *cftr*^pd1049^ allele (null mutants). This clearly demonstrates that the forskolin+IBMX stimulated KV fluid secretion is mediated by CFTR. The next step was to expose the *pkd2*-morphants to the forskolin+IBMX treatment. We expected them to have at least, 3.2 times the volume of WT siblings, i.e. 1.6 times from the *pkd2*-augMO effect plus 1.6 times from the forskolin+IBMX effect. Interestingly, a synergistic effect between these two factors was observed with forskolin+IBMX treated *pkd2*-morphants presenting, on average, KVs with 3.6 times the volume of WT siblings (forskolin+IBMX treated *pkd2*-morphants KV^volume^=215×10^3^±70×10^3^ µm^3^ vs *pkd2*-morphants KV^volume^=92×10^3^±23×10^3^ µm^3^; *P*<0.0001) (Fig. 2B,J,K). This indicated that these two factors synergistically activate CFTR.

Is this crosstalk between the lack of Polycystin-2 and the stimulation of CFTR a cell autonomous process? To answer this question we targeted the *pkd2*-knockdown into the DFCs (Essner et al., 2005). This time we assessed the KV volume by scanning the whole organ in embryos immunostained for actin cystoskeleton at the 8–10 s.s. Our data clearly show that increasing amounts of morpholino lead to a gradual dilation of the KV (Fig. S3). Indeed, while observing a very small effect when injecting 4 ng, a significant dilation was observed when using 9 ng. These data supports the hypothesis that, although being expressed in KV surrounding tissues, it is the specific knockdown of Polycystin-2 in the KV cells that leads to CFTR stimulation. The possible mosaic uptake of the morpholino, a recurrent problem in this approach (Essner et al., 2005), may justify the requirement of such high amounts of *pkd2*-augMO to induce the KV dilation.

### Inflation dynamics of the *pkd2-*morphant KV

**Fig. 4.**
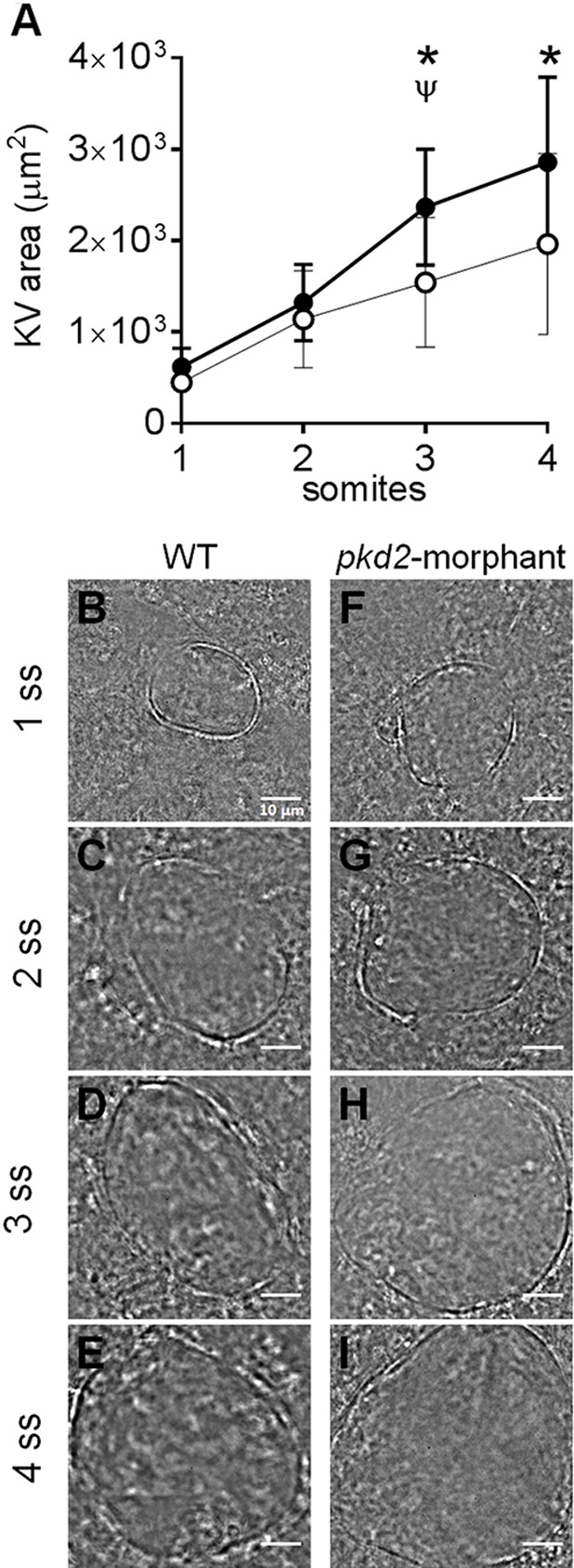
**KV inflation live-dynamics.** (A) KV luminal area of *pkd2*-morphants and WT embryos measured along development from 1 to 4 s.s. For each time point mean±s.d. are indicated. Number of tested embryos: WT, *n=*11; *pkd2*-morphant, *n=*8. ^ψ^*P*<0.05, significantly different from the previous time point in *pkd2*-morphants; *P<0.05, significantly different from WT at the corresponding time point. (B-I) Bright field images captured for the same embryo along development are shown for the most representative WT (B-E) and *pkd2*-morphant (F-I). Scale bars: 10 µm.

Navis et al. showed that the apical expression of CFTR in the cells facing the lumen of the KV was detected from the very beginning of its lumen formation, i.e. from 1 s.s. (Navis et al., 2013). So, we next evaluated at what stage did the volume started to increase upon the Polycystin-2 knockdown. For that we followed the live-dynamics of the KV inflation in *pkd2*-morphants and WT embryos (Fig. 4). We observed that in both cases the KV inflation rate was the same until the 2 s.s. (Fig. 4A,B,C,F,G). It was only at the 3 s.s. that the rate of inflation of the KVs from *pkd2*-morphants increased and became significantly different from WT embryos (Fig. 4A,D,H). This suggests that the overstimulation of CFTR upon the knockdown of Polycystin-2 occurs early in the KV morphogenesis, but not immediately after its formation.

## DISCUSSION

Through largely unknown mechanisms, ADPKD cystogenesis involves two major steps: the formation of the cyst, which requires aberrant proliferation of tubule epithelial cells; and the cyst inflation, that requires marked transepithelial fluid secretion towards the cyst lumen (Wallace, 2011).

Numerous studies using cellular (Li et al., 2004, 2012; Reif et al., 2011; Yuajit et al., 2013) or mouse models for ADPKD (Blazer-Yost et al., 2010; Yang et al., 2008; Yuajit et al., 2014) have proved the key role of CFTR in the cyst inflation process. This can be slowed down by the use of CFTR-interfering molecules, such as: steviol and its derivatives that inhibit CFTR and promote its proteasome-mediated degradation (Yuajit et al., 2013, 2014); CFTRinh-172 (Li et al., 2004), the CFTR blocker used in our experiments; and pioglitazone, an agonist of the peroxisome proliferator-activated receptor γ which reduces the CFTR mRNA levels (Blazer-Yost et al., 2010). The overexpression of the commonest mutation associated to CF, F508del, where the protein fails in reaching the cell membrane, was also shown to slow *in vitro* cystogenesis (Li et al., 2012).

**Fig. 5.**
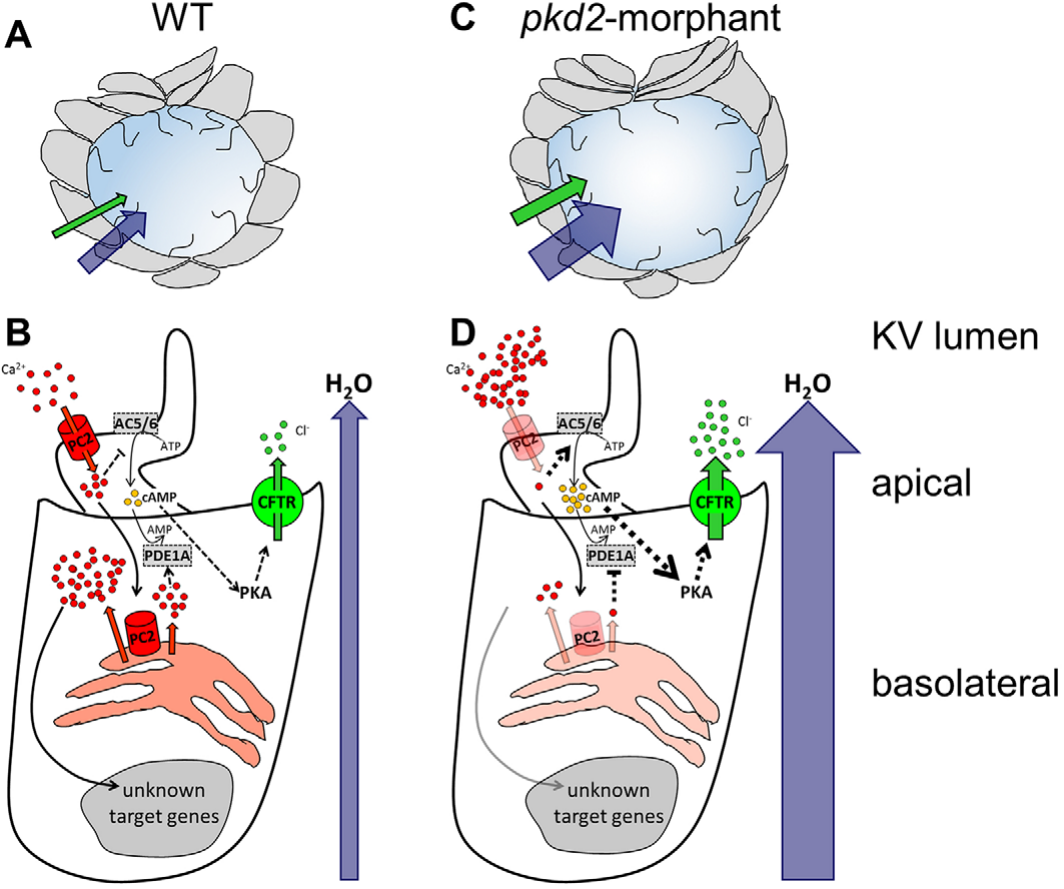
**The zebrafish KV as a model organ for CFTR stimulation in ADPKD cysts.** (A,B) In WT embryos, the KV inflation is ensured by the CFTR-mediated transport of Cl^−^ and by the subsequent movement of water towards the organ lumen (A). In KV epithelial cells (B), Polycystin-2 (PC2) located at the cilia membrane allows the entrance of Ca^2+^ when stimulated by the luminal fluid-flow. This ciliary wave activates the Ca^2+^ release from the ER pools, in a Polycystin-2-dependent manner, initiating a Ca^2+^-signaling of unknown effectors. Through inhibition of adenylyl cyclases 5 and 6 (AC5/AC6) and activation of phosphodiesterase 1A (PDE1A), the Ca^2+^ transients maintain the basal intracellular levels of cAMP required for the normal rate of CFTR activity. (C,D) Mimicking ADPKD cysts, the *pkd2*-knockdown enhances CFTR-mediated ion and fluid secretion into the KV, resulting in its significant enlargement (C). The reduced Ca^2+^ oscillations are expected to activate AC5/AC6 and inhibit PDE1A, raising the intracellular levels of cAMP and, thus, driving the overstimulation of CFTR (D). Ca^2+^ and Polycystin-2 (red); Cl^−^ and CFTR (green); cAMP (yellow); H_2_O (blue); AC5, AC6, and PDE1A (grey). Full black arrows – known activations; dashed lines and arrows – expected inhibitions/activations; line and arrow widths are proportional to the expected level of activation.

The available ADPKD model systems, although useful in testing the effectiveness of those molecules, are limited in the study of the precise mechanisms by which the lack of Polycystin-2 leads to CFTR stimulation. In our perspective, such model system must fulfil some requirements: (1) it should have a fluid-filled vesicular structure resembling the architecture of ADPKD renal cysts; (2) it should be lined with ciliated cells, since cells facing the lumen of ADPKD kidney cysts are ciliated (Thomson et al., 2003; Xu et al., 2007); (3) these cells should endogenously express both CFTR and Polycystin-2 and the system should allow the easy knockdown of each of them; (4) under normal conditions, it should exhibit a fluid-flow induced Ca^2+^-signaling mediated by Polycystin-2, which should be impaired in the absence of Polysystin-2; (5) its lumen inflation should be dependent on CFTR; and, most importantly (6) the lack of Polycystin-2 should result in larger volumes through stimulation of CFTR. Based on our findings and hypotheses we propose the zebrafish KV to be that model-organ (Fig. 5).

### KV has a cyst-like structure

KV is a fluid-filled enclosed cavity, lined by monociliated epithelial cells. It forms during early somitogenesis at the posterior end of the embryo from the DFCs cluster and is transiently present from 1 to 14 somites (Essner et al., 2005). The relative simplicity and experimental accessibility of KV compared with other organs undergoing *de novo* lumen formation make it an attractive model-organ. Indeed, during the time window of KV existence, embryos are fully transparent allowing the easy access to the KV volume by confocal live-microscopy of *ras*:GFP or *foxji1*:GFP transgenic embryos.

### KV cells express both CFTR and Polycystin-2

At the 10–11 s.s., we detected both *cftr* and *pkd2* transcripts in the KV region. Regarding *pkd2* expression, our results are in line with previous studies that reported marked expression of *pkd2* transcripts in the zebrafish DFCs during gastrulation, that become more diffuse as these cells form the KV (Bisgrove et al., 2005; Schottenfeld et al., 2007). Regarding *cftr* expression, our data are in line with Navis et al., who have reported an enrichment of these transcripts in zebrafish KV at the 3 s.s. that also turned to be more diffuse as somitogenesis proceeded (Navis et al., 2013). At the protein level, those authors showed the apical expression of CFTR in cells lining the KV (Navis et al., 2013) and we show here, that these cells also express Polycystin-2.

### The intracellular distribution of Polycystin-2 supports its involvement in the KV fluid-flow induced Ca^2+^-signalling

We found Polycystin-2 distributed through the cytoplasm, many times clustered near the nuclei. This is in agreement with the literature that describes ER as the major location for Polycystin-2 within zebrafish, mouse and human cells (Cai et al., 1999; Fu et al., 2008; Gainullin et al., 2015; Hofherr et al., 2014; Pazour et al., 2002). Indeed, Polycystin-2 has a conserved ER retrieval motif, a cluster of acidic amino acids at its C-terminal, also present in the zebrafish orthologue (Fu et al., 2008). It has been suggested, however, that a balanced subcellular compartmentalization of endogenous Polycystin-2 is important for the proper functioning of the zebrafish KV in the left-right axis determination (Fu et al., 2008). In accordance, we also detected Polycystin-2 along all KV cilia, recapitulating the expression pattern observed in medaka KV (Kamura et al., 2011), zebrafish pronephros (Obara et al., 2006) and mouse and human renal cells (Gainullin et al., 2015; Pazour et al., 2002).

The intracellular distribution of Polycystin-2 corroborates its involvement in Ca^2+^ transients recently demonstrated to occur within zebrafish KV (Yuan et al., 2015). Accordingly, as KV cilia acquire motility become capable of generating a fluid-flow within the lumen of the organ. This fluid-flow triggers Polycystin-2-dependent intraciliary Ca^2+^ oscillations which are subsequently transduced into cytosolic Ca^2+^ waves (Yuan et al., 2015), as represented in Fig. 5B. In this way, a Ca^2+^-signaling pathway of unknown effectors is initiated to control the subsequent events necessary for the correct organ positioning in the embryo (Yuan et al., 2015). This brings the KV epithelium closer to the normal renal tubular epithelium. Indeed, the urine flow triggers a Polycystin-2-mediated ciliary Ca^2+^ signaling in the human tubular epithelial cells, which is essential for the maintenance and differentiation of the renal tissue. *In vitro* cell based experiments have demonstrated that in response to a fluid shear stress, a Polycystin-2-dependent ciliary Ca^2+^ influx occurs, driving a Ca^2+^ release from ER stores (Jin et al., 2014; Nauli et al., 2003; Praetorius and Spring, 2001; Xu et al., 2007). We should highlight, however, that we are not bringing KV cilia close to kidney cilia in terms of their structure or function. Indeed, these differ in many ways. Cilia from normal kidney cells are immotile and do not generate flow. Although not much is known about cilia from kidney cysts, it is plausible that these differ from the primary cilia present in the normal tubular epithelia (Thomson et al., 2003; Xu et al., 2007).

### *cftr* and *pkd2* expression are easily knocked down in KV cells

Supporting the efficient knockdown of CFTR and Polycystin-2, both the *cftr*-augMO and the *pkd2*-augMO induced heart position defects, phenocopying the previously described *cftr* and *cup^−/−^* zebrafish mutants, respectively (Navis et al., 2013; Schottenfeld et al., 2007). To our knowledge, laterality problems caused by the lack of CFTR were never reported in CF patients, possibly because the use of CFTR in the left-right organizer might be a teleost specific mechanism.

Mirroring kidney cysts, Polycystin-2 was no longer detected along *pkd2*-morphants KV cilia. Indeed, the cilia of ADPKD kidney cysts epithelial cells lack the expression of Polycystin-1, -2 or both (Xu et al., 2007). Additionally, *pkd2*-morphants showed a marked reduction in the cytoplasmic levels of the immunodetected Polycystin-2. A more accurate quantification of the knockdown efficiency was hampered by the fact that all the commercially available antibodies against mammalian Polycystin-2 that we have tested failed in detecting the protein by western blot. Nevertheless, as represented in Fig. 5D, it was recently shown that the knockdown of Polycystin-2 with the same *pkd2*-augMO used in our experiments suppresses, in number and amplitude, the intraciliary Ca^2+^ oscillations and the subsequent cytoplasmic Ca^2+^ waves in KV-lining cells (Yuan et al., 2015).

Our attempts to quantify the *cftr*-knockdown failed as none of the tested commercially available antibodies against human CFTR cross-reacted with the zebrafish protein, either in whole-mount immunostaining or by western blotting. Nevertheless, the lack of CFTR in *cftr*-morphants was easily observed by the failure of KV expansion at the 10–11 s.s. This is in line with the fact that in mutants homozygous for *cftr*^pd1049^ allele (null mutation) KV inflation does not occur and those homozygous for the *cftr*^pd1048^ allele (missense mutation) have small KVs (Navis et al., 2013). Knowing that the absence of CFTR does not affect the apical-basal polarization, nor the ciliogenesis of the KV cells (Navis et al., 2013), our data clearly supports its essential role in the KV inflation. CFTR transports Cl^−^ towards the lumen of KV driving the movement of water across the KV epithelium (Fig. 5A and B).

### Reduced levels of Polycystin-2 lead to CFTR-dependent over-inflation of the KV

The relevance of the KV as a model for ADPKD cyst inflation is highlighted by the fact that *pkd2*-morphants have significantly larger KVs than WT siblings. While not involving cell proliferation, this KV dilation is due to more fluid secretion into the KV's lumen (Fig. 5C). According to Compagnon et al. (2014), more fluid secretion into the KV drives cell shape modifications by enhancing the intraluminal pressure. As expected, *pkd2*-morphants exhibited significant differences in the shape of the cells facing the KV lumen midplan, both anteriorly and posteriorly. Finally, we pharmacologically demonstrate that, in a cell autonomous manner, the KV enlargement observed in *pkd2*-morphants is mediated by CFTR activity. Indeed, the volume of the *pkd2*-morphant KVs was rescued by CFTR inhibition and was synergistically enlarged by further stimulating CFTR.

We first used ouabain to block the CFTR activity as this was used by others to prove that the KV expansion requires CFTR (Navis et al., 2013). However, more than CFTR activity, ouabain affects the ion transport in general, since it is a Na^+^/K^+^-ATPase inhibitor (Lingrel, 2010). We, thus, tested the effect of CFTRinh-172. Our data showed a strong effect of both molecules in rescuing the KV lumen expansion of *pkd2*-morphants to volumes equivalent or, even, smaller than WT embryos. These data strengthen the use of KV as model system for the stimulation of CFTR in ADPKD because, as already mentioned, CFTRinh-172 also slows down *in vitro* cyst enlargement (Li et al., 2004). In the future, it will be interesting to test the effect of other molecules that have been shown to be effective in slowing down cystogenesis in ADPKD cellular and animal models (Blazer-Yost et al., 2010; Li et al., 2004, 2012; Yang et al., 2008; Yuajit et al., 2013, 2014). These experiments are even more appealing given the fact that zebrafish CFTR responds to many pharmacological activators and inhibitors of human CFTR activity (Bagnat et al., 2010).

To potentiate CFTR, we used forskolin+IBMX. These molecules are known to raise the cellular cAMP levels. Indeed, forskolin stimulates all adenylyl cyclases (ACs) and IBMX globally inhibits phosphodiesterases (PDEs). Therefore, as the activation of CFTR demands its prior phosphorylation by PKA and, in turn, PKA activity depends on the intracellular levels of cAMP, higher cAMP levels lead to the activation of CFTR. Under our experimental conditions, we verified that these two drugs act synergistically with the knockdown of Polycystin-2 towards enlarging the KV luminal volume. Our data suggest that just like forskolin+IBMX, the knockdown of Polycystin-2 stimulates CFTR in KV epithelial cells by raising the intracellular levels of cAMP. This brings the KV model-organ closer to kidney cysts. Indeed, renal cAMP levels are elevated in ADPKD mice models and in human cyst epithelial cells and have been suggested as one possible reason for the stimulation of CFTR (Pinto et al., 2012; Spirli et al., 2012; Wallace, 2011). Several evidences point to persistent synthesis and less degradation of cAMP in ADPKD tissues (Pinto et al., 2012; Torres et al., 2012; Wallace, 2011). A possible explanation arises from the activation of the Ca^2+^-inhibited AC5 and AC6 and the inhibition of the Ca^2+^-calmodulin-dependent PDE1 because of the reduced Ca^2+^ levels found in ADPKD cells (Wallace, 2011). Supporting our model system, the knockdown of PDE1A aggravated the body curvature and the renal phenotype of *pkd2*-morphant zebrafish larvae, whereas the PDE1A overexpression partially rescued both (Sussman et al., 2014). We have recently performed a tissue specific microarray analysis (our unpublished observations) which showed that KV epithelial cells express endogenously AC5, AC6a and AC6b and PDE1A. Taken all together, we propose that as in kidney cysts, in KV cells the drop of the intracellular Ca^2+^ levels caused by the knockdown of Polycystin-2, leads to the activation of AC5 and AC6 and to the inhibition of PDE1A, raising the levels of cAMP and, thus, activating CFTR (Fig. 5D). Future studies are required to further support this mechanistic overlap between KV development and renal cystogenesis.

An important question for the ADPKD field is the time point at which CFTR is activated during cystogenesis and whether this is different when the disease-causing mutation affects *pkd1* or *pkd2*. Indeed, *pkd1* mutations are associated with significantly more severe disease, triggering cystogenesis earlier in patients' lives (Cornec-Le Gall et al., 2013). Although we cannot solve this problem with our model yet, we hope to contribute to it in a near future. As in kidney cells, Polycystin-2 in zebrafish KV cells is expected to complex with a Polycystin-1 paralogue, the Polycystin-1-like-1. This is the Polycystin-2 partner in medaka KV (Kamura et al., 2011) and in mouse node (Field et al., 2011). Once this missing piece in the puzzle has been found, we will be able to compare the KV inflation dynamics of *pkd1l1*-morphants with that of *pkd2*-morphants. So far, we were able to identify the time point at which CFTR turns to be overstimulated in *pkd2*-morphants, which was at the 3 s.s.

In conclusion, we show good evidence to consider zebrafish KV an appropriate model system to study the mechanisms involved in the stimulation of CFTR upon the lack of Polycystin-2. Allowing the measurement of its volume as a live readout, it offers an excellent *in vivo* model for screening compounds and genes that may slow down cyst enlargement through CFTR inhibition.

## MATERIALS AND METHODS

### Fish strains

WT, *ras*:GFP transgenic (Cooper et al., 2005) and *cup^tc321^* mutant zebrafish lines, all of AB background, were maintained at 28°C. The latter was outcrossed with *foxj1a*:GFP transgenic line of Tupfel long fin background and, from these, only *cup^+/−^*;*foxj1a*:GFP were kept. Embryos obtained from incrosses were incubated in E3 medium at 25°C or 28°C and staged as described elsewhere (Kimmel et al., 1995). All procedures were approved by the Portuguese Direcção Geral de Veterinária and Instituto Gulbenkian de Ciência (IGC) ethics committee.

### MO microinjections

The knockdown of *pkd2* and *cftr* was induced by injecting one-cell stage embryos with 1.8 and 1.6 ng of the translational blocking morpholinos *pkd2*-augMO (Schottenfeld et al., 2007; Sun et al., 2004) and *cftr*-augMO (5′-TCCTCCACAGGTGATCTCTGCATCC-3′), respectively. 1.8 ng of a *pkd2-*mismatchMO was used as control. All morpholinos were purchased from Gene Tools LLC (Philomath, USA). To target the knockdown of Polycystin-2 to DFCs, *pkd2*-augMO was diluted in 1:4 (v/v) rhodamine-dextran *M*r 10,000 solution (Sigma-Aldrich, USA) and injected (4 ng and 9 ng) into the yolk in mid-blastula stage embryos as previously described (Essner et al., 2005).

### Heart laterality scoring

The heart jogging was evaluated at 30 hpf by observing the embryos from their ventral side, using a stereoscope (SMZ745, Nikon Corporation, Japan).

### *In situ* hybridization

Whole-mount *in situ* hybridizations were performed as before (Sampaio et al., 2014). A fragment of *cftr* and *pkd2* zebrafish genes (ENSDART00000020412 and ENSDART00000020412, respectively) were amplified by PCR. The anti-sense RNA probes were transcribed using SP6 RNA polymerase.

### Immunofluorescence on whole-mount embryos

Dechorionated 10–11 s.s. embryos and those grown until 36 hpf (incubated in 0.1 mM 1-phenyl-2-thiourea to avoid pigmentation) were fixed in 80:20 (v/v) methanol:DMSO for 1 min and rehydrated in sequential incubations in crescent dilutions of methanol in PBS. The latter ones were further incubated with Proteinase K (10 µg/ml) for 15 min at RT. After permeabilization and blocking, embryos were incubated overnight at 4°C with anti-Polycystin-2 polyclonal antibody (1:200; GTX113802, GeneTex, USA) and, subsequently, with anti-acetylated α-tubulin (1:400; T7451, Sigma) and anti-γ-tubulin (1:100; C-20, Santa Cruz Biotechnology, Germany) monoclonal antibodies. Some embryos were incubated with Alexa Fluor 488 phalloidin (1:100; Molecular Probes, USA) for actin cytoskeleton evaluation. Alexa Fluor 488, 546 or 647 conjugated secondary antibodies (1:500; Molecular Probes) were used. Nuclei were stained with DAPI. Flat-mounted embryos were analyzed with confocal fluorescent microscopy (Zeiss LSM710) and their whole KVs were scanned with *z*-sections of 0.5 µm. Movies were analyzed using ImageJ. Selected stacks were used to count the number of KV cilia.

### Live-imaging in zebrafish KV

Embryos were mounted in a 2% (w/v) agarose mold and covered with E3 medium. For volumes evaluation, the whole KVs of 10–11 s.s. *ras*:GFP transgenic embryos were scanned by confocal-live microscopy, with *z*-sections of 0.5 µm and acquisition rate lower than 1 frame per second. These stacks were also used to count the number of KV-lining cells. To evaluate the KV-inflation dynamics, the KV midplan of AB embryos was followed along development, from 1 to 4 s.s., by light microscopy (Nikon Eclipse Ti-U inverted microscope), under a 100×/1.30 NA oil immersion objective lens and recorded the images with a FASTCAM-MC2 camera (Photron Europe Limited, UK) controlled with Photron FASTCAM Viewer software. Using the ImageJ plugin Measure Stack, the KV was delineated and its luminal area was measured in all focal planes. The volume resulted from the sum of the measurements of all focal planes. The average values are referred to as KV^volume^.

### Cell shape evaluation

Selected stacks of embryos immunostained for actin cytoskeleton were analyzed in Amira for 3D (FEI, USA) for cellular measurements (length, width and height) in the KV midplane. Cell apical surface area was given by the product of cellular width and height values.

### Pharmacological treatments

Stock solutions: 10 mM forskolin (Sigma), 100 mM IBMX (3-isobutyl-1-methylxanthine; Sigma) and 10 mM CFTRinh-172 (CF Foundation Therapeuticals), all in DMSO; and 1 mM ouabain (Sigma) in water. Embryos were treated at 10 μM forskolin, 40 μM IBMX, 30 µM CFTRinh-172 and 5 μM ouabain in E3 from 6 s.s. onwards.

### Statistics

Differences were analyzed for statistical significance using Student's *t*-test in Prism (Graphpad, USA), being considered as statistically significant when *P*<0.05. Results are expressed as mean±standard deviations (s.d.) of *n* observations.
